# Mothers’ Experiences about Decisions to Use Children’s Proton Beam Therapy

**DOI:** 10.3390/children8040274

**Published:** 2021-04-02

**Authors:** Noriko Ozawa, Rieko Fukuzawa, Kayuri Furuya

**Affiliations:** 1 Faculty of Medicine, University of Tsukuba, Ibaraki 305-8577, Japan; rkishi1@md.tsukuba.ac.jp; 2 Faculty of Global Nursing, Iryo Sosei University, Chiba 277-0803, Japan; furuya.kayuri@isu.ac.jp

**Keywords:** proton beam therapy, radiation therapy, pediatric nursing, parent, experience

## Abstract

Recently, proton beam therapy has been recommended in radiation therapy for child-hood cancer. However, facilities for children are limited, and parents who choose this treatment for their children face a variety of challenges. This study reveals mothers’ experiences about the decision to use the aforementioned therapy. A semi-structured interview was conducted with 16 mothers of children who received proton beam therapy in Japan, and a grounded theory approach was adopted. The results revealed that mothers were very worried about late complications concerning their children due to radiation. While the mothers strongly expected proton beam therapy to reduce the risk of late complications, they felt uncertainty and anxiety throughout the entire decision-making process. Despite having to deal with their feelings, they had to transfer to another hospital and prepare support for their children to begin treatment, and this put a lot of strain on them. From decision-making to start of treatment, these emotional fluctuations and the need for psychological support became apparent.

## 1. Introduction

In Japan, 2000 to 2500 children are diagnosed with cancer every year [1]. They receive multidisciplinary therapy, including surgery, chemotherapy, and radiation therapy. About 80% of these children can be cured through the progress of treatment [2,3,4]. While survival rates have improved, survivors suffering from late complications are increasing [5,6,7]. In recent years, treatment that considers not only lifesaving measures but also the children’s quality of life (QOL) has become a necessity [4,8].

Radiation therapy is a necessary treatment for saving children’s lives, but X-rays or γ-rays tend to cause serious late complications, such as secondary cancer and hormone abnormalities [5]. Therefore, a treatment that has received attention because of fewer late-stage complications is proton beam therapy. Proton beam therapy can reduce the effects on normal tissues [9]. It can reduce late complications and therefore is expected to improve QOL of survivors [10]. Proton beam therapy is recommended for children if they are indicated for proton therapy [11,12].

However, there are some difficulties for children receiving proton beam therapy. A child’s cancer not only affects the child but also the entire family. Parents are stressed by supporting sick children [13,14]. If their child needs to receive radiation therapy in addition to chemotherapy or surgery, they must acquire new information about radiation therapy, organize it, and prepare themselves to support their children. Furthermore, they work hard to support their child and ensure they can be safely treated [15,16]. Among the difficulties of children’s proton beam therapy is the limited number of institutions that offer this treatment. Japan has the second largest number of facilities (11) after the United States, but less than half of those facilities are equipped to treat children [17]. Therefore, many children who will receive proton beam therapy often live far from the medical facilities where they could be treated. They are frequently transferred from one hospital to another to receive this treatment [18,19]. In addition, mothers of children receiving proton beam therapy may also have to stay away from their family and familiar health care professionals to accompany their children. They need to build relationships with health care professionals in the new environment. Therefore, it has been reported that there is a need for more support, but mothers receive less social support after this transfer [20]. It is necessary to consider a support system that accounts for the mother’s psychological condition.

Despite this situation, few studies have focused on the mothers of children receiving proton beam therapy. In particular, no study has examined the mother’s psychological state, including the decision-making process about how mothers choose proton beam therapy and, subsequently, how they felt about the treatment. Their psychological situation influences their attitudes toward subsequent treatment [21]. The decision-making process and how these changes influence their psychological situations need to be clarified for the nursing care of the mother. Therefore, this study attempted to clarify what mothers experience in the course of their decision-making process to start proton beam therapy.

## 2. Materials and Methods

This qualitative inductive research was an inductive study using the grounded theory approach (GTA).

This study received approval from the clinical research ethics review committee of the target facility (approval no. H-28-201). Data collection was conducted from January 2017 to January 2019. The attending physician explained the purpose of the study to the mother of the child undergoing proton beam therapy. When consent was obtained, we met them in person to explain the research. The participants were given a written and verbal explanation informing them that participation was voluntary and that consent could be withdrawn. Written informed consent was obtained from all participants.

The study was conducted at one hospital in Japan that has the longest record of providing pediatric proton beam therapy for children. It is a facility that has been working on proton therapy for children since 1983, before the insurance was applied in 2016. Participants included the mothers of children undergoing proton beam therapy. Mothers of children who received treatment for the purpose of palliative treatment or those who came to Japan for treatment were excluded.

The principal investigator conducted a semi-structured interview with the participants. The interview was recorded on an IC recorder, and the facial expressions, tears, and gestures of the participants were recorded in a field note. The interviewer was a nurse with 10 years of experience of taking care of the children and their families while undergoing proton beam therapy.

In this study, we used grounded theory [22] to investigate how mothers interact with their surroundings in decision-making and, as a result, how they approach the treatment. Grounded theory provides an approach for defining a phenomenon as a process resulting from the interrelationship of meanings (symbols) that are generated from perceptions, actions, and interactions with subjects [23,24,25]. Grounded theory’s fundamental theory is symbolic interactionism [26]. Awareness of structure (conditions), process (actions/interactions), and consequences (outcomes), known as a paradigm, are thought of as key factors for understanding the structure [22,23,27].

Our analysis consisted of the following steps [23]:

### 2.1. Open Coding

(1) We created text through field notes and tape transcriptions.
(2) We read the text and made the segment.
(3) We extracted the properties and dimensions from the segment and gave them label names. The property is a concept representing a viewpoint while the dimension indicates what the dimension is from a given perspective. The basis for describing dimensions as “high” and “low” were expressions such as “very,” “not too much,” and gestures and facial expressions.
(4) The labels were combined, and categories were named after checking for similarities between properties and dimensions.

### 2.2. Axial Coding

(1) We used a paradigm framework of situations, actions/interactions, and consequences to create categories from the perspective of when, where, what to do, how, and the result.
(2) We examined the association between categories from the perspective of how the context changes depending on the combination of properties and dimensions, and how it promotes actions/interactions with other categories. We have created a related diagram that shows the direction of change with arrows and how it changes between categories. This related diagram shows the process that the participants followed in the phenomenon. So once the association diagram, we confirmed that all the participants’ storylines were explained. After that, one central category was selected and named as the phenomenon.

The sample size for qualitative research varies depending on the subject and purpose. Grounded theory approach calls for saturation to determine a sample size, and theoretical sampling was used to help identify the next purposive set of children and procedures [22]. Saturation was judged by interpretability.

For the reliability of the analysis, after one person analyzed the data, a second researcher confirmed the coding of content for all processes. If we had any discrepancies, we went back to the previous process and rechecked the data, discussed the discrepancy, and agreed to a final outcome.

## 3. Results

We contacted eighteen mothers, sixteen of which participated in this study. Two mothers declined to be involved, because they were experiencing anxiety that they could not talk about their feelings yet. Their age ranged from 25 to 45 years (Mean = 32.3 years, SD = 6.4). To receive proton beam therapy, all children needed to transfer from another district for about two months. Fifteen mothers had transferred with their children. One mother had to travel for about two hours by car everyday while her child was in treatment. All the mothers who participated in this study had a husband, none of the mothers lived with their children’s grandparents. In all cases, the home was far from the proton therapy facility, and the father stayed home, continuing to work and take care of other children. When possible, or on holidays, they sometimes visited the children receiving proton therapy and their mothers. On the other days, they usually communicate with each other by phone or other means. Table 1 shows the attributes such as the diagnosis name of the child. Eleven children had siblings. There were eight boys and eight girls, and their age ranged from 1 to 12 years (Mean 4.2 years). All had received chemotherapy prior to proton beam therapy and all had undergone surgery. Seven children were scheduled to end treatment after proton therapy. Nine children had scheduled chemotherapy or surgery after proton beam therapy. Interview times ranged from 53 min to 82 min (Mean 61 min).

### 3.1. Category Descriptions

There are nine categories in the phenomenon. Below, the italicized text represents participant quotations translated from Japanese into English. Table 2 shows the definitions, corresponding properties, and dimensions of the nine categories.

#### 3.1.1. Conflict between Lifesaving and Future Worries

Some mothers had been told that whether their children needed radiation therapy depended on the results of surgery and chemotherapy. The more they knew about radiation therapy, the more they found out about higher risks of late complications in their children. The mothers had supported children’s chemotherapy and surgery, hoping that their children would not need radiation therapy. They felt hopeless when they were told by their doctors that they needed radiation therapy as a result of treatments. The mothers understood that saving their children’s lives was paramount, but they worried about future late complications and wondered if they should receive radiation therapy. They were “conflicted between lifesaving and future worries.”

*Interview, P4: As a parent, honestly, I had thought that even if our child would have some disabilities, we (parents) need to save [the] child’s life. However, when I realized that my child needed to receive radiation, I felt the desire that my child should not have any disabilities. In addition to saving children’s lives at this point, I strongly hoped that they would stay healthy for five, ten, or thirty years. The desire to avoid radiation therapy grew stronger, but there was no other option to save my child.*

#### 3.1.2. Belief That Only Proton Beam Therapy Is Available

Mothers who were hesitant to apply X-rays, which have a high risk of side effects and late complications, had gained information about proton beam therapy on their own, even if they had only received information about X-ray treatment from their doctors. Only six mothers had gotten specific information from their doctors about proton beam therapy from the beginning. Ten mothers looked for information on proton beam therapy on their own. Knowing the benefits, they increased their expectations that proton beam therapy would not cause late complications. This intensified their ‘‘belief that only proton beam therapy is available’’, and they had hoped to receive it. Despite receiving a second opinion at the facility where they were actually treated and being told that there was a risk of late complications and side effects with proton therapy, their expectations for proton beam therapy had been increasing day by day.

#### 3.1.3. Overcoming the Difficulties Associated with the Transfer

After choosing proton beam therapy, they are faced with the difficulty of transferring to another hospital. Even if their expectations for proton beam therapy were high, mothers felt difficulties supporting their children in the new environment and treatment. They had to try to ‘‘overcome the difficulties associated with the transfer.’’ For example, in the 11 case of mothers with other children who had to leave home with the child who had to undergo treatment, some were worried about the situation in which they had to leave their other children at home without their mother. They had to coordinate the care of their other children to accompany their child in the transfer. Some mothers felt the pressure that they had to support their children themselves at the new hospital without being at home and having familiar medical staff. Therefore, some mothers had been reluctant to change hospitals and worried about changes in the environment. However, they had prepared to support their children with their family because they had wanted to make a commitment to proton beam therapy.

*Interview, P10: I hesitated to transfer to another hospital. If I accompanied the child to another hospital, it would put more stress on the other children. Since the diagnosis of my child’s illness, I had been spending time supporting the child. The other children had become psychologically unstable. I wanted to play many roles for both the ill child and their sibling as a mother, but I have limitations. At that time, my husband and my mother had supported me.*

*Interview, P2: I was worried that I had to deal with the problem alone without my husband if my child’s physical condition changed after the transfer. I felt responsible for it strongly and I was anxious.*

#### 3.1.4. X-rays Are Inevitable

Some mothers whose children had been determined to need radiation therapy at the first tumor diagnosis had thought that late complications were unavoidable. They were less ‘‘conflicted between saving the life of their child and future worries.’’ If they had not received a strong recommendation for proton beam therapy from their doctors, they would have eventually chosen X-rays, comparing the benefits of proton therapy with the difficulty of “overcoming metastases”.

#### 3.1.5. Increased Anxiety about the Child’s Future

After deciding on proton therapy and preparing for a transfer, the mothers finally moved to the hospital with their children. After the move, they received treatment explanations again. At that time, doctors once again explained the side effects and risk of late complications. Although it had been explained before, the mothers realized that they had avoided thinking about it and rather focused on the expected the benefits. Once again, they worried about the child’s future. The explanations were provided almost after separating with their family and familiar medical staff, and thus, many mothers had received these explanations alone. They were shocked when their support had diminished, so they had become more and more worried.

*Interview, P8: After the transfer, I was convinced that I could no longer get rid of my anxieties regarding my child’s late complication. I had always been worried about the recurrence of cancer in my child and secondary cancers. Even if the worry of recurrence is over, the worry of secondary cancer continues forever. My anxiety became even stronger when the treatment was about to begin.*

#### 3.1.6. Anxiety That the Disease May Progress

After the transfer, it would take some time, about one week, to adjust to the transfer and prepare for the irradiation. Even though the preparation had been quick, one mother had felt that the time it had taken was exceptionally long. She was worried that her child’s tumor might become larger during their preparation period.

*Interview, P5: It had taken about a week to make a mask for proton beam therapy. I understood, but [I was] worried that the tumor would progress. That time was very long for me.*

#### 3.1.7. Worry about the Treatment Choice until the Last Minute

Mothers, in particular those who had the option of choosing another way, worried about the treatment choice until just before treatment began.

*Interview, P5: I had decided [on] my child’s proton beam therapy, but then I got lost [in] my decision. My child had another option [of] undergoing surgery. I had been worried and thought [about it] until the last minute.*

#### 3.1.8. Switching from Feelings of Anxiety to Motivation to Receive Treatment

The phenomenon had two outcomes and this category is one of these outcomes. Even though the anxiety of some mothers was increasing, they thought that this was not the time to be concerned about their anxiety because the proton beam therapy for their children was imminent. The mothers switched from feelings of anxiety about the treatment to focusing on supporting their child in the treatment.

*Interview, P4: There were a lot of anxieties, but the treatment was about to start and there was a lot to do. So I switched to not thinking about anxiety.*

*Interview, P5: I thought that although I would worry again after the irradiation ended, for the time being, I thought I had to finish the irradiation. It was my primary first goal to finish proton therapy for my child’s safety. Perhaps, in a sense, since there were a lot of things to do about my child’s irradiation every day, I didn’t have time to think about my anxieties. So during the irradiation it might have been easier for me than before the irradiation.*

#### 3.1.9. Accepting Anxiety and Having a Positive Feeling about Treatment

This category is the second outcome. Some mothers had been concerned about their anxiety. However, they had shared their anxiety with their families and new medical staff and had looked back on their treatment decision-making process. With the belief that their choice of proton beam therapy was the best course of action, some were able to lower their anxieties and felt positive motivation for the treatment again.

*Interview, P10: After the transfer of the child, I had been told by a doctor about the side effects, and I became more anxious, so I called my husband and talked about the doctor’s explanation and my feelings. Actually, I wished we had heard the doctor’s explanation together, but my husband had been taking care of our other children, so he couldn’t come. On the phone, we had discussed my worries about it. Looking back, we noticed that we had known that it is not an all-purpose treatment, and could notice this way was best for our child again. This way we could have tried to do it with a positive attitude.*

*Interview, P8: I contacted my mother. She told me to face my child’s present life rather than to worry about the future. I had been thinking about the child’s future. But then I was convinced that the most important thing is to save my child’s life. I remembered my intention and could feel positive about the treatment again.*

### 3.2. Description of the Process Pattern

**Figure 1:**
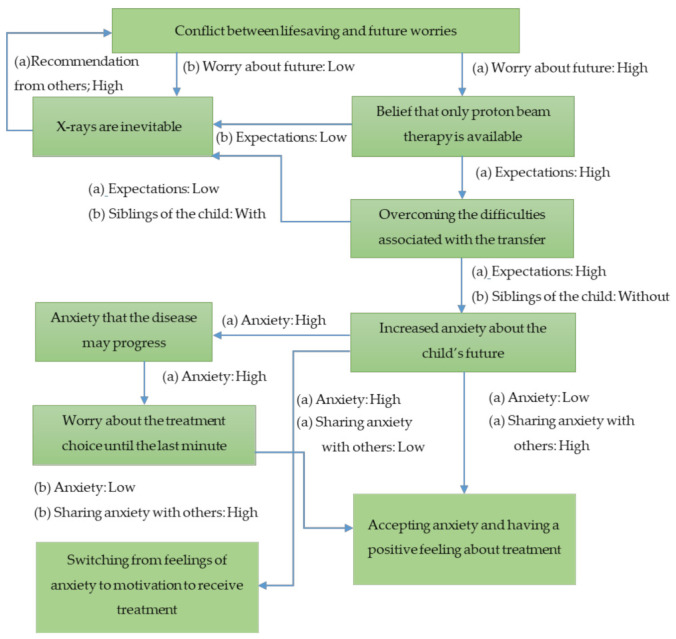
Process of mothers’ feelings about proton beam therapy. Boxes indicate the category. Arrows represent the processes between the categories. Phrase(s) next to arrow: the dimension experienced in the process.

Figure 1 shows the process of the mother’s experience in making decisions about proton beam therapy. The box shows the category along with the associated properties and dimensions (see Table 1 for categories, properties, and dimensions). In this figure, the arrows between the boxes show how categories relate to each other through specific properties and dimensions. In general, thematic map categories are associated with several properties and dimensions [23]. However, for space and readability, Figure 1 shows only the key dimensions.

Many mothers were worried about their child’s future complications and wanted to avoid radiation therapy as much as possible. They focused on the benefits of proton beam therapy, which can be expected to reduce late complications, and raised their expectations for it. The greater the concern about the child’s future and the lack of access to medical professionals, the higher this expectation. The mother knew that proton therapy also had a risk of late complications, but she unknowingly avoided thinking about them.

The difficulty of transferring to another hospital for proton therapy was more likely to occur when the children have young siblings or when the mother herself was afraid of changes in the environment. They were trying to overcome difficulties by collaborating with other family members and raising expectations for treatment. It turns out that some mothers would choose X-rays at the hospitals where they have already been treated if the difficulties for transferring were higher than the expectations for proton beam therapy.

In fact, after the child had been transferred to another hospital for treatment, they had the opportunity to receive explanations again, which again increased their anxieties about the future of the child. The more they had worried before, the greater their anxieties. Moreover, mothers who were worried about being placed in a new environment away from their families and familiar medical staff felt lonely and had increased anxieties.

There were mothers who had been worried about their own decision until the last minute, but eventually, all tried to support the child’s proton beam therapy.

Some mothers were willing to take treatment with the feeling that they had no choice but to set aside their anxiety and concentrate on treatment. They were trying to think about anxieties again when the treatment was over. These mothers were trying to focus on the smooth completion of their child’s proton therapy, which was their only goal. On the other hand, some mothers overcame their anxieties and took a positive approach to treatment. The difference in this process is that mothers could express their feelings to distant family members and newly met medical staff, and in the process, they also looked back on their decision-making process. It was influenced by the fact that they remembered that they had made the choice for their child and they could increase their affirmation of proton beam therapy.

## 4. Discussion

The main category in the results of this study was “Increased anxiety about the children’s future.” It will be important to discuss why mothers have increased anxiety about the future of their children just before treatment and what kind of support they need.

In this study, it became clear that the mothers of children receiving proton therapy had increased uncertainty about the child’s future, which in turn had an effect on the mother’s psychological status. Uncertainty about what will happen, what the effect of the disease or treatment is significant [28,29]. The management of uncertainty has been shown to be a challenge for adaptation30,31. Previous studies have shown that childhood cancer treatment is complex and that it is difficult to predict the future of the child and the course of the treatment and that parents are more likely to have uncertainties as a result. Furthermore, it has been shown that their uncertainty increases if the treatment content was changed after the diagnosis [30,31,32]. In addition, research shows that children who receive radiation therapy are at high risk for late complications, their future is difficult to predict, with a high level of uncertainty of mothers [15]. In particular, the mothers of the children receiving proton beam therapy in this study made this decision to reduce the anxiety related to uncertainty of their child’s future. In many cases, the medical staff gave little information about proton beam therapy to the mothers. The mothers obtained such information themselves and coped with their concerns of uncertainty by raising their expectations for the treatment, which is important in decision-making [33]. However, their anxieties increased again when they faced risks just before their children received treatment. The increasing anxiety had been affected by the biased information obtained by themselves, who had tried to not see the information about the late complications of their child’s actual treatment. One of the factors that increase uncertainty is the lack of information [34], providing appropriate information to mothers of children undergoing proton beam therapy is one of the important means of support. Previous studies have shown that intervention in mother’s uncertainty can reduce her anxiety. In addition, recently, focus on patient and family engagement has led to interest in shared decision making (SDM) in pediatric [35]. SDM aims to engage patients and clinicians in a partnership to make medical decisions that are supported by the best available evidence and aligned with patient’s values, preferences, and treatment goals [36,37] This study showed the importance and difficulty of SDM in proton beam therapy, because of the necessity of transfer. In this treatment, particularly, it is clear that we have to support the family so that various information can be continuously obtained. Furthermore, we have to assess continuously how the family themselves perceive that information, even since before the transfer to after the transfer.

After the transfer, many mothers were separated from other family members, and they felt pressured by the thought that they have to support their children alone. Mothers of children who receive proton beam therapy must support their children in new treatments [15,16] while also addressing their concerns about their distant families [20]. In addition, they are far from familiar hospitals and need to build relationships with new health care professionals. There are many anxieties in the situation in which there is also a decrease in the social support they had previously received [20]. On the other hand, mothers revealed that despite being anxious, they also tried to positively provide support to their children during the treatment process. The reflections on the decision-making process had prompted mothers to support their children with a positive feeling. It was important that they derive meaning from their decisions as the result of thinking hard about their children’s future. Uncertainty is not only a negative effect, but in a way, can also lead to family growth and family strength [28]. Parents’ reflections on their child’s treatment choices, regardless of the treatment outcome, may affect the way parents deal with the treatment experience [38]. The mother of a child receiving proton beam therapy thinks about the future of her child and has to overcome many difficult aspects, such as a hospital transfer. Reflecting on and recognizing these challenges that they accomplished for their children will be their strength [39,40]. Thus, it is important to share their anxiety with their families or medical staff and to support a reflection and an understanding of the meaning of their decision-making process. In particular, since treatment will occur across two medical institutions, coordination between medical facilities is necessary to ensure the care of mothers and their children even if their hospital changes. In order to provide adequate care to the family, have to share with multiple facilities and medical staffs, the things that the family values, how the family overcame the treatment, such as chemotherapy or surgery, and the decision-making process.

Finally, important support is interdisciplinary psychological support for the family system. Parents of children with childhood cancer are trying to address the uncertainty about their child’s illness and treatment and adapt to changing family roles and relationships with healthcare professionals [41,42]. Further, these situations often adversely affect family psychology. The study found that mothers had a lot of anxiety due to the characteristics of radiation therapy at high risk of late complications and the further change in family roles due to the need for transfer. Through the mother’s experience, it is clear that the entire family of children receiving proton therapy is variously affected. It is necessary to consider intervention to the family by psychologists/psycho-oncologists such as “Family Psych Oncological Counseling” and “Phasic Family Therapy,” [43] is described. These interventions may improve active cooperation between family members, patients, and medical teams, and also support families who have to adapt to the uncertainties of proton therapy and changes in their relationships with their families and healthcare professionals.

### Limitations

Proton beam therapy for children is a relatively new treatment. Since the number of children with cancer who receive proton therapy is limited, this study has a small sample. In addition, this study took place at one facility in Japan which offers proton beam therapy to pediatric cancer patient, because the facilities are limited. It is necessary to further increase the number of target people and facilities to confirm new patterns in the future. Furthermore, as our study only targeted mothers, it will be necessary to focus on other family members as well to understand the experiences of the family. While we focused on the decision-making process, in the future, it will be necessary to focus on treatment and after treatment for a further description of the mother’s experience.

Despite the above limitations, phenomena such as how mothers’ feelings change during the decision-making process for proton beam therapy have not been clarified until now. The findings of this study may help health care professionals understand families’ difficulties that result from the need to transfer to a hospital that provides proton beam therapy for children.

## 5. Conclusions

We investigated mothers’ experiences about their decision-making relating to proton beam therapy for their children. The results showed that mothers chose this treatment to reduce their risks about the future of their children, however, their anxieties increased just before treatment and they felt some difficulties in the decision-making process. The needs for intervention in maternal uncertainty, SDM, and family counseling were suggested. To that end, it is important to cooperate with other facilities and medical professionals, provide appropriate information to mothers and family, and provide continuous psychology support. Additionally, it is important to provide supports to help these mothers and family become aware of their strengths.

## Figures and Tables

**Table 1:** Children’s attributes (n = 16).

Age	
0–3	8
4–6	5
7–10	2
10–12	1
Gender	
Male	8
Female	8
Siblings	
Yes	11
No	5
Diagnosis	
Medulloblastoma	4
Rhabdomyosarcoma	7
Ependymoma	3
Retinoblastoma	1
Yolk sac tumor	1
Recurrence	
Yes	2
No	14

**Table 2:** Categories of “Increased anxiety about the children’s future” and their definitions.

Category	Brief Definition	Main Properties	Range of Dimensions
Conflict between lifesaving and future worries	Radiation is necessary to save the child’s life, but there are risks for later complications in the future. They had concerns about the decision for undergoing radiation therapy.	Degree of worry about the future Degree of hesitation to receive treatment	High (a); Low (b) High (a); Low (b)
X-rays are inevitable	They thought they had no choice but to choose X-ray therapy to save their children.	Degree of recommendation from doctors and family	High (a)
Belief that only proton beam therapy is available	They strengthened their belief that the only treatment children should receive is proton beam therapy.	Degree of expectations	High (a); Low (b)
Overcoming the difficulties associated with the transfer	They had difficulties in transferring for treatment. They were worried about taking care of other siblings left at home without their mother, and they were not confident in supporting their child’s treatment in a new environment. They wanted to do it for their child, and they overcame difficulties with their families.	Degree of expectation Siblings of the child Degree of own anxiety about the new environment	High (a); Low (b) With (a); Without (b) Low(a); High (b)
Anxiety that the disease may progress	They felt that the preparation period for starting irradiation was too long. They vacillated in their decision with other options.	Feeling that the length of time is too long Presence of other options	High (a); Low (b) Have (a); Nothing (b)
Increased anxiety about the child’s future	Prior to starting the irradiation, their anxiety about the child’s future increased. With a doctor’s explanation, they had to contemplate the risks of later complications again, which they had been trying not to think about.	Degree of anxiety they had before Degree of sharing anxiety with others	High (a); Low (b) High (a); Low (b)
Worrying about the treatment choice until the last minute	Although they had decided to receive proton beam therapy, once its start was imminent, they were in a fog with other treatment options.	Degree of hesitation	High (a)
Switching from feelings of anxiety to motivation to receive treatment	They were not free of anxiety but felt that they should not think about their anxiety, to support their child’s treatment. They thought the important thing at the moment was to concentrate on the treatment because the treatment would be completed safely.	Degree of anxiety for the child’s future	
Accepting anxiety and having a positive feeling about treatment	They could accept the anxieties and think that these feelings would be inevitable to some extent, through looking back on their decision-making with their families and medical staff. They could try to support their child’s treatment with positive feelings.	Degree of anxiety for the child’s future	

## Data Availability

The data in this study is a survey of parents in hospital in Japan and is very sensitive, including information on in relation to legal minors. In the surveyed hospital, disclosure of raw data is not allowed even if anonymous.
